# Endothelial SR-B1 is dispensable for thermogenesis but promotes selective cholesterol uptake in brown adipose tissue

**DOI:** 10.1016/j.jlr.2025.100894

**Published:** 2025-09-03

**Authors:** Kimberley M. Hurkmans, Markus Heine, Franz Rinninger, Michelle Y. Jaeckstein, Chieko Mineo, Philip W. Shaul, Joerg Heeren

**Affiliations:** 1 Department of Biochemistry and Molecular Cell Biology, University Medical Center Hamburg-Eppendorf, Hamburg, Germany; 2 Center for Pulmonary and Vascular Biology, Department of Pediatrics, University of Texas Southwestern Medical Center, Dallas, TX, USA

**Keywords:** adipose tissue, brown, scavenger receptors, cholesterol/metabolism, fatty acid transport, lipoprotein/receptors, lipoprotein/metabolism, adaptive thermogenesis, heart, triglycerides

## Abstract

In an interplay with parenchymal cells of metabolically active organs, such as heart and adipose tissues, vascular endothelial cells are important for the regulation of nutrient uptake and organ-specific energy metabolism. Based on high expression of the scavenger receptor class B type I (SR-B1) in capillary endothelial cells of white adipose tissue and brown adipose tissue (BAT), we proposed a functional role for this receptor in lipid handling and adaptive thermogenesis. To address this hypothesis, we generated mice with an endothelial-specific KO of SR-B1 and performed metabolic turnover and indirect calorimetry studies in response to environmental cues, such as cold exposure and high-fat diet feeding. Compared with control littermates, endothelial-specific SR-B1 KO mice had substantially lower SR-B1 mRNA and protein levels in heart, skeletal muscle, BAT, and white adipose tissue but not in liver, indicating that SR-B1 is primarily expressed by endothelial cells in peripheral organs. We did not detect major differences in gene expression of thermogenic and lipid-handling genes, energy expenditure assessed by indirect calorimetry, or clearance of metabolic tracers for glucose and triglycerides between endothelial SR-B1 KO mice and controls under basal conditions, thermogenic activation, or high-fat diet feeding. However, consistent with the importance of SR-B1 expression by hepatocytes for HDL metabolism, mice lacking endothelial SR-B1 had lower selective cholesterol uptake in the heart and BAT compared with control littermates. We conclude that endothelial SR-B1 is not essential for adaptive thermogenesis and handling of triglyceride-rich lipoproteins, but it is involved in regulating cholesterol homeostasis in the heart and BAT.

Adipose tissue is a heterogeneous organ and is important for whole-body energy homeostasis. The two types of adipose tissue are white adipose tissue (WAT) and brown adipose tissue (BAT). The main function of WAT is to store energy in the form of triglycerides and to release FFAs under catabolic conditions such as fasting. BAT can also internalize and accumulate significant amounts of triglycerides that are stored in multilocular lipid droplets. However, unlike white adipocytes, thermogenic brown adipocytes burn fatty acids to produce heat to maintain core body temperature in colder environments. This process, known as adaptive thermogenesis, is primarily activated by BAT innervating sympathetic nerves, which, by releasing norepinephrine, activate β2 and/or β3-adrenergic receptors expressed by thermogenic adipocytes (1, 2). The subsequent intracellular signaling cascade results in the hydrolysis of triglycerides by intracellular lipases (3), and the released fatty acids are then imported into mitochondria to fuel beta oxidation (4). In addition to serving as energy substrates, liberated fatty acids activate the uncoupling protein 1 (UCP1) that is specifically expressed by thermogenic adipocytes. This unique protein uncouples the proton gradient generated at the inner mitochondrial membrane from ATP production, a process that leads to the generation of heat (1, 5).

In addition to intracellularly liberated fatty acids, activated BAT also takes up large amounts of glucose and lipids from the circulation to replenish lipid energy stores (6, 7). The main source of energy comes from triglyceride-rich lipoproteins (TRLs), which are produced by the gut or the liver, depending on the nutritional status. To provide FFAs for thermogenesis, TRLs have to be hydrolyzed by LPL, the gatekeeper enzyme bound to glycosylphosphatidylinositol-anchored HDL-binding protein 1 (GPIHBP1) on the vascular endothelium (8). After hydrolysis, TRL-derived FFAs are transferred via the endothelial barrier and are internalized by thermogenic adipocytes of BAT. Although the detailed mechanisms of transendothelial transport are still uncertain, various lipid transporters have been described as being critically involved in fatty acid transport (9). One of these transporters is the protein cluster of differentiation 36 (CD36), which, next to scavenger receptor class B type I (SR-B1) and the lysosomal integral membrane protein II, belongs to the class B scavenger receptor family (10, 11, 12). CD36 is a long-chain fatty acid transporter and plays an important role in thermogenesis (13, 14, 15). Full-body CD36 KO mice are unable to maintain core body temperature in response to cold exposure, which can be explained by substantially lower lipid uptake in BAT (7). Interestingly, under conditions of mild cold exposure, the uptake of TRL-derived fatty acids into the BAT is similar between wild-type and CD36-deficient mice (16, 17), arguing for alternative transport processes that may play a role in endothelial lipid uptake. One potential candidate is SR-B1, which is known to be involved in mediating selective cholesteryl ester uptake into parenchymal cells of liver and adrenals, thereby promoting reverse cholesterol transport and steroid hormone production, respectively (18). In the context of the current study, it is important to note that SR-B1 expressed by arterial endothelial cells can promote the uptake of whole lipoprotein particles (19). This study demonstrated that LDL particles undergo endothelial transcytosis via SR-B1, ultimately leading to arterial cholesterol deposition and atherosclerosis. Similarly, we observed the endocytosis of whole TRL particles into endothelial cells of activated BAT (16). Furthermore, we were able to show that intracellular TRL processing along the endolysosomal route is important for cold-induced recruitment of thermogenic adipocytes and efficient energy expenditure (16). However, whether SR-B1 has a role in lipoprotein uptake and lipid handling in capillary endothelial cells in metabolically active organs in vivo is unknown.

In the current study, we aimed to unravel the metabolic role of endothelial SR-B1 for nutrient uptake in the context of adaptive thermogenesis and high-fat diet (HFD) feeding. Gene and protein expression analysis showed high expression of SR-B1 in endothelial cells of adipose tissues. No major phenotypic differences or alterations in metabolic parameters were observed in mice lacking SR-B1 specifically in endothelial cells. Furthermore, metabolic studies conducted at different ambient temperatures revealed that endothelial SR-B1 is not essential for intraluminal TRL processing, endocytosis of whole lipoprotein particles, and energy expenditure. Metabolic HDL tracer studies demonstrated that selective cholesterol uptake into the heart and BAT is mediated by endothelial SR-B1, suggesting that this scavenger receptor is involved in cholesterol homeostasis of peripheral metabolically active organs.

## Materials and methods

### Animals

Mice with a specific endothelial deletion of SR-B1 and their control littermates were studied (19). *Scarb1*^fl/fl^ mice have loxP sites inserted in intron 1 and intron 3 of the *Scarb1* gene. Heterozygous *Scarb1*^fl/+^ mice were crossed with *Cdh5*^Cre+^ mice to generate mice with an endothelial-specific KO of SR-B1 (*Scarb1*^fl/fl^-*Cdh5*^Cre+^) and control littermates (*Scarb1*^fl/fl^-*Cdh5*^Cre−^). Age-matched male and female mice (10–24 weeks old) were used for the experiments unless stated otherwise. Mice were housed individually during the experiment with wood bedding in a light- and temperature-controlled facility with a 12 h/12 h dark regime. Mice were fed a standard laboratory chow diet ad libitum (19.10% protein, 4% fat, 6% fiber, from Altromin Spezialfutter GmbH&Co, Germany) or an HFD for 4 weeks (26.1% protein, 25.5% carbohydrates, 6.4% fiber, 34.8% fat, 6.4% mineral, 0.4% vitamins, 0.25% cholesterol from Research Diets, D14010701) prior to the start of the experiment. At the end of the experiments, body weight was measured and blood glucose was determined using an Accu-Chek Aviva (Roche). Unless stated otherwise, mice were sacrificed by a lethal dose of a mix of ketamine (180 mg/kg) and xylazine (24 mg/kg) in 0.9% NaCl. Blood samples were collected in syringes containing 0.5 M EDTA by cardiac puncture, and subsequently, the animals were perfused with ice-cold PBS containing 10 U ml^−1^ heparin. Harvested tissues were snap frozen in liquid nitrogen for quantitative PCR and Western blot analysis. Frozen tissues were stored at −80°C. Animal studies with these mice were carried out in Hamburg and were approved by the Animal Welfare Officers at the University Medical Center Hamburg-Eppendorf and the Behörde für Gesundheit und Verbraucherschutz Hamburg.

### Human WAT samples

At the Department of General, Visceral, and Thoracic Surgery, University Medical Center Hamburg-Eppendorf, samples from subcutaneous and visceral adipose tissues were harvested in patients undergoing bariatric surgery. All participants signed an informed consent. The study was approved by the Ethics Committee of the Hamburg Chamber of Physicians (PV4889) and was conducted in accordance with the Declaration of Helsinki.

### Indirect calorimetry

A week before the start of the experiment, for continuous monitoring of core body temperature, mice underwent surgery to transplant a transponder (STARR Life Sciences) in the intraperitoneal cavity. For metabolic studies, mice were housed in metabolic cages (Sable Systems Europe GmbH, Germany), and the system was operated according to the manufacturer’s guidelines. Mice were acclimated to the metabolic cages for 2 days at 22°C prior to recording and then acclimated to cold from 30°C to 6°C by decreasing the temperature by 6°C every day at 7 am. Consumption of O_2_, production of CO_2_, food intake, water intake, core body temperature, and activity were measured for 7 consecutive days. Respiratory exchange ratio (RER) was calculated by dividing CO_2_ production (VCO_2_) by O_2_ consumption (VO_2_) (VCO_2_/VO_2_) and energy expenditure by using the Weir equation (kcal per day = 1.440 (3.9 ∗ VO_2_ + 1.1 ∗ VCO_2_) (20). On the day of sacrifice, mice were fasted for 4 h in the morning and received a CL-316,243 (Tocris, 0.2 mg ml^−1^ in 0.9 w/v % NaCl) subcutaneous injection (1 μg per g body weight) and were sacrificed by cervical dislocation for tissue collection 3 h later. To determine metabolic parameters of *Scarb1*^*fl/fl*^-Cdh5^Cre+^ and *Scarb1*^*fl/fl*^-Cdh5^Cre−^ mice in an unchallenged state (22°C, chow fed), fat mass, lean mass, and water mass were measured using the EchoMRI machine (EchoMRI™). Afterward, mice were kept in metabolic cages (Sable Systems Europe GmbH, Germany) for 5 days at 22°C. For comparison of mice with mild versus strong BAT activation, half of the mice were kept at room temperature (22°C = mild cold exposure for mice), and the other half was put into the cold (6°C = robust cold exposure for mice) for 24 h. Mice were fasted for 4 h in the morning before sacrifice.

### Plasma triglyceride and cholesterol determination

Blood samples were centrifuged for 5 min, 10,000 *g*, and subsequently, plasma was collected. For plasma triglyceride and cholesterol determination, 5 μl of plasma was pipetted in duplicate into a 96-well plate and analyzed using commercial kits (DiaSys Diagnostic Systems GmbH).

### Lipoprotein profiling

Plasma (120 μl per mouse) was collected after a 4-h fast from male mice (age 12 weeks) maintained on standard chow or shifted to a cholesterol-rich HFD for 4 weeks. For lipoprotein profiling by fast-pressure liquid chromatography (FPLC), samples were analyzed individually. Plasma from four mice per genotype and diet was applied to a Superose 6 Increase 10/300 GL column (GE Healthcare) and eluted with PBS/1 mM EDTA at 0.5 ml min^−1^. Thirty fractions of 0.5 ml were collected and assayed enzymatically for total cholesterol (DiaSys Diagnostic Systems GmbH). Genotype effects were tested by two-way repeated-measures ANOVA (genotype × fraction) followed by Šidák post hoc comparison; *P* < 0.05 was considered significant.

### Oral glucose fat tolerance test

Half of the mice were kept at room temperature, and the other half were exposed to cold (6°C) for 24 h. Mice were fasted for 4 h in the morning. Two hours prior to sacrifice, they received an oral gavage containing radiolabeled [^3^H]-triolein and [^14^C]-deoxyglucose (each at 1.9 MBq/kg body weight), along with unlabeled triolein (4.3 g/kg body weight) and glucose (2 g/kg body weight) as described (16, 17). Organs were dissolved in Solvable™ (PerkinElmer) on a shaker at 60°C overnight; radioactivity was then measured using a Tri-Carb® 2810 TR Liquid Scintillation Analyzer (PerkinElmer).

### Magnetic-activated cell sorting

To isolate different cell types from BAT and WAT, magnetic-activated cell sorting (MACS®) analysis was performed as described previously (21). For murine BAT and WAT, tissues were pooled separately from four mice per pool, resulting in n = 3 per genotype. Subsequently, tissues were digested with collagenase D (Sigma). Mature adipocyte (mA) fraction was collected by collecting the floating fraction after low-speed centrifugation. Remaining fractions were collected with the use of magnetic beads (Miltenyi Biotec GmbH, Germany) and LS columns (Miltenyi Biotec GmbH, Germany). First, the CD11b^+^ cells reflecting the macrophage fraction and then the CD31^+^ cells representing the endothelial cell fraction were isolated. For human visceral and subcutaneous adipose samples, tissues were minced and digested for 30 min at 37°C in isolation buffer (123 mM NaCl, 5 mM KCl, 1.3 mM CaCl_2_, 5 mM glucose, 100 mM Hepes; pH: 7.4) containing 600 U/ml collagenase II and 1.5% BSA. The following magnetic microbeads were used: mouse/human CD11b microbeads (Miltenyi; 130-049-601, 10 μl beads/107 cells), human CD31 microbead kit (Miltenyi; 130-091-935, 10 μl beads/10^7^ cells and 10 μl FcR blocking reagent/107 cells), and mouse CD31 microbeads (Miltenyi; 130-097-418, 10 μl beads/10^7^ cells). Cell fractions were pelleted and resuspended in TRIzol® reagent for RNA extraction.

### Gene expression analysis

RNA was isolated using the Nucleo Spin® RNA (Macherey-Nagel™, Germany) according to the manufacturer’s protocol. RNA content was measured using NanoPhotometer® N60 (IMPLEN, Germany), and subsequently, 400 ng of RNA isolated from organs was transcribed to complementary DNA using the High-Capacity Complementary DNA Reverse Transcription Kit (Applied Biosystems). From isolated cells obtained in the MACS® experiment, 100 ng of RNA was transcribed. Gene expression was measured by Quantstudio™ 5 Real-Time PCR Systems (ThermoFisher Scientific), and the following TaqMan® Assay-on-demand primer sets were used: *Tbp*: Mm00446973_m1, *Scarb1*: Mm01198172_m1, *Ucp1*: Mm00494069_m1, *Elovl3:* Mm00468164_m1, *Ppargc1a*: Mm00447183_m1, *Fasn*: Mm00662319_m1, *Dio2*: Mm00515664_m1, *Lpl*: Mm00434764_m1, *Cd36*: Mm00432403_m1, *Scarb2*: Mm00446977_m1, *Glut1*: Mm00441480_m1, *Glut4*: Mm01245502_m1, *Ldlr*: Mm00440169_m1; *Lrp1*: Mm00464608_m1, *Vldlr*: Mm00443281_m1, *Emr1*: Mm00802530_m1, *Gpihbp1*: Mm01205849_g1, *36B4:* Hs99999902_m1, *ADIPOQ:* Hs00605917_m1, *GPIHBP1:* Hs01564843_m1, *EMR1:* Hs00173562_m1, and *SCARB1:* Hs00969821_m1, *VLDLR:* Hs00182461_m1. Cycling parameters were as follows: 1 cycle of 95°C for 10 min, 40 cycles of 95°C for 15 s then 60°C for 60 s, followed by melt curve analysis. Gene copy number was calculated with the formula (10^((Ct-35)/-3.3219)^), and gene expression was normalized for copy number of *Tbp or 36B4*. Normalized values could be used to calculate the relative values where the values for *Scarb1*^fl/fl^-*Cdh5*^Cre−^ were set to 1.

### Western blot

Total lysates were prepared by homogenizing the tissues in RIPA buffer supplemented with protease inhibitors (Roche) and phosphatase inhibitors (Sigma). Samples (20 μg per lane) were separated by SDS-PAGE and transferred to a nitrocellulose membrane overnight. Ponceau staining was performed to ensure equal loading. The blots were blocked in 5% milk in Tris-buffered saline with Tween-20 (TBST) for 1 h at room temperature. After washing with TBST, blots were incubated with primary antibody in 5% BSA in TBST overnight at 4°C. Primary antibodies used were SR-B1 (ab217318, 1:1,000 dilution) and AKT (cs9272, 1:1,000 dilution). After further washing with TBST, blots were incubated with secondary antibody diluted in 5% milk in TBST for 1 h at room temperature. Blots were developed on Amersham Imager 600 using luminol (Sigma; A4865) and para-hydroxycoumarinic acid-based (Sigma; C9008) chemiluminescence substrate. The secondary antibody used was HRP-conjugated goat anti-rabbit (AB_2307391; Jackson ImmunoResearch Laboratories, Inc, 1:5,000 dilution). Quantification of the bands was performed using Image Studio Lite (version 5.2.5), and protein expression was normalized to AKT. From the normalized values, the relative values were calculated where the values for *Scarb1*^fl/fl^-*Cdh5*^Cre−^ were set to 1.

### HDL turnover

HDL was isolated by sequential ultracentrifugation (d = 1.063–1.21 g/ml) as described by Havel *et al.* (22) from C57BL/6J WT mice, which were fasted 4 h before blood withdrawal. HDL was then double-labeled with ^125^I-tyramine cellobiose (^125^I-TC) in the apolipoprotein moiety and with ^3^H-cholesteryl oleoyl ether (^3^H-CEt) in the lipoprotein core as described before (23). The faster plasma decay of HDL-associated ^3^H-CEt compared with ^125^I-TC indicates selective HDL cholesterol ether clearance from the plasma compartment. Because TC remains stably crosslinked to apolipoproteins, any dissociated label is rapidly cleared renally and does not accumulate in organs (23). Briefly, human plasma CETP was used to introduce ^3^H-CEt into ^125^I-TC-HDL by exchange from donor liposomal particles containing ^3^H-CEt. The final ^125^I-TC-/^3^H-CEt-HDL particles were dialyzed against PBS (pH 7.4, 4°C) with added 1 mM EDTA. Subsequently, ^125^I-TC-/^3^H-CEt-HDL (30 mg HDL protein per mouse; ca. 39 kBq ^125^I-TC and 33 kBq ^3^H-CEt, respectively) was injected into the tail vein of 4 h fasted mice for analysis of plasma decay and organ uptake of radiolabeled HDL. Blood samples were collected at given times after injection: 10 min, 30 min; 2, 5, 9, 22, and 24 h. While plasma aliquots and tissues were directly assayed for ^125^I radioactivity, ^3^H-radioactivity was analyzed by scintillation counting after lipid extraction using the method of Dole (24). Plasma fractional catabolic rates (plasma-FCRs) were determined for the two tracers per mouse. The uptake of the HDL-associated tracers was determined in the following organs: liver, kidney, heart, spleen, intestine, and interscapular BAT (iBAT). The fraction of total tracer uptake attributed to a specific organ was calculated as the radioactivity recovered in that organ divided by the total radioactivity recovered from all tissues and carcass. To allow comparison of the specific activities of various tissues in HDL internalization and to directly compare the rates of uptake of the apo component and the CE moiety of HDL, the data are expressed as organ fractional catabolic rates (organ-FCRs). These rates are calculated as follows: (organ-FCR in tissue X) = (plasma-FCR) × (fraction [%] of total body tracer recovery in tissue X). This organ-FCR represents the fraction of the plasma pool of either HDL tracer cleared by an organ per hour. ^125^I-TC represents the uptake of HDL holoparticles by tissues. Selective HDL CEt uptake is calculated as the difference in organ-FCR between [^3^H]CEt and ^125^I-TC.

### Statistical analysis

Results are expressed as mean ± SEM. Outliers were identified using GraphPad Prism (version 10 for Windows) (GraphPad Software) via the ROUT method with a Q cutoff value of 1%. When the outliers were identified in basal body characteristics (body weight, plasma cholesterol and triglyceride levels, and organ weights), the outlier was removed from all subsequent analyses. All other statistical analyses were performed with RStudio (version 2023.12.1+402) (RStudio) with the following packages: readxl, tidyverse, car, ggpubr, and rstatix. Data were tested for normal distribution and homogeneity of variances. When parametric assumptions were met, a Student’s *t*-test was used for comparisons between two groups; otherwise, a two-way ANOVA was performed. When a nonparametric test was required, a Wilcoxon rank-sum test was used for two-group comparisons. A *P* value <0.05 was considered significant. Statistical differences are indicated with different letters.

## Results

### SR-B1 is mainly expressed by vascular endothelial cells of adipose tissues

**Fig. 1:**
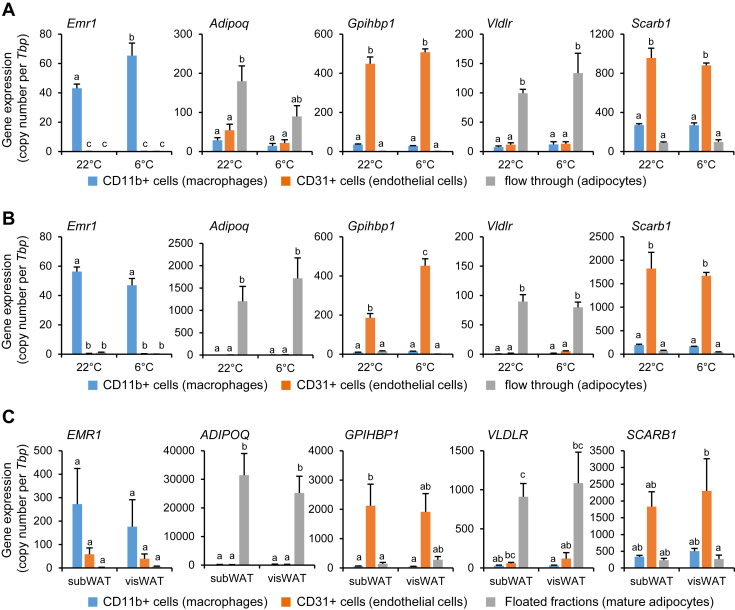
Impact of endothelial SR-B1 on metabolic parameters and energy metabolism. A and B: Wild-type mice were housed at 22°C or at 6°C. Using MACS® technology, CD11b+ macrophages), CD31+ endothelial cells, and flow through (adipocytes) were isolated from (A) BAT and (B) iWAT (n = 4). Expression levels of specific markers for macrophages (*Emr1*), adipocytes (*Adipoq*), and endothelial cells (*Gpihbp1*) as well as *Vldlr* and *Scarb1* were determined by qPCR. The mean of copy numbers of four experiments ± SEM is shown. C: Floating mAs, CD11b+ (macrophages), and CD31+ endothelial cells were isolated from subcutaneous (subWAT) and visceral (visWAT) fat depots of three individuals undergoing bariatric surgery using MACS® technology. Expression levels of specific macrophage (*EMR1*), adipocyte (*ADIPOQ*), and endothelial cell markers (*GPIHBP1*) as well as *VLDLR* and *SCARB1* were determined using specific TaqMan® probes for the indicated genes. The mean of copy numbers ± SEM is shown. Different letters indicate statistical difference *P* < 0.05.

CD36 and the VLDL receptor (VLDLR) have been shown to be involved in lipoprotein processing and fatty acid uptake in thermogenically activated adipose tissues (17, 25). Moreover, recently, we showed that endothelial cells of WAT and BAT can internalize entire TRLs, a process partly dependent on CD36 (16). As SR-B1 promotes LDL uptake into endothelial cells of the arteries (19), we here asked the question whether SR-B1 contributes to the processing of lipoproteins in the capillary lumen of metabolically active tissues. To study the cellular distribution, we isolated endothelial cells, macrophages, and adipocytes by MACS® of murine adipose tissues. To confirm the cellular identity of the isolated fractions, we measured genes specific for the endothelial cells (*Gpihbp1*), myeloid cells (*Emr1*), and adipocytes (*Adipoq*) in BAT (Fig. 1A) and WAT (Fig. 1B). In line with the potential function for endothelial lipoprotein processing in the capillaries of adipose tissues, we detected high SR-B1 (encoded by *Scarb1*) expression levels in vascular endothelial cells of BAT (Fig. 1A) and WAT (Fig. 1B) of both warm- and cold-exposed mice. In contrast, *Vldlr* expression was predominantly detected in adipocytes. To validate cell type-specific expression in human adipose tissues, we isolated macrophages, endothelial cells, and adipocytes from human visceral and subcutaneous fat depots by a similar MACS®-based approach. In line with data generated in mice, we found high expression of SR-B1 in CD31-positive endothelial fractions, whereas *VLDLR* was detected predominantly in human adipocytes (Fig. 1C). Overall, these data suggest that SR-B1 expressed by endothelial cells could play a role in the lipid metabolism of the adipose tissue of both mice and humans.

**Fig. 2:**
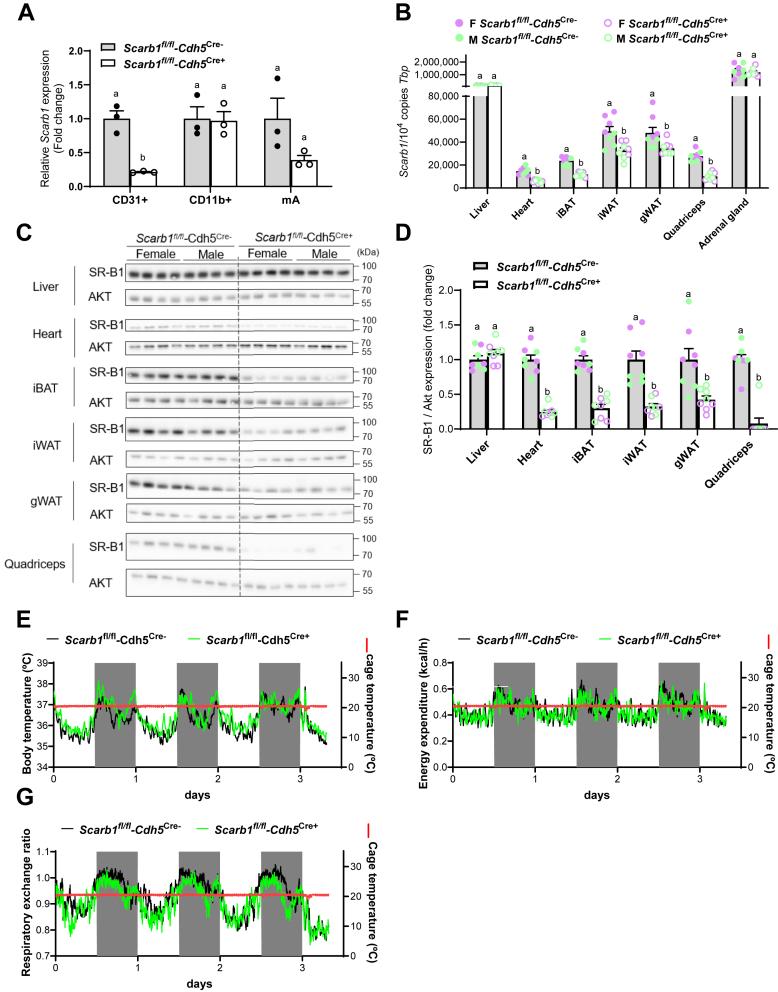
Impact of endothelial SR-B1 on metabolic parameters and energy metabolism. A: Female *Scarb1*^*fl/fl*^*-Cdh5*^*Cre+*^ and *Scarb1*^*fl/fl*^*-Cdh5*^*Cre*−^ mice were housed at room temperature and fed a chow diet. MACS®-based isolation of endothelial cells (CD31+), myeloid cells (CD11b+), and mAs of BAT extracts was performed, and *Scarb1* expression was determined by qPCR in the three cell fractions from iBAT of *Scarb1*^*fl/fl*^*-Cdh5*^*Cre+*^ and *Scarb1*^*fl/fl*^*-Cdh5*^*Cre*−^. Data are normalized to *Tbp* expression and presented as mean values ± SEM, and *Scarb1*^*fl/fl*^*-Cdh5*^*Cre*−^ group was set to 1. B–D: Female (pink dots) and male (green dots) *Scarb1*^*fl/fl*^*-Cdh5*^*Cre+*^ and *Scarb1*^*fl/fl*^*-Cdh5*^*Cre*−^ mice (n = 4 per genotype, per sex) were housed at room temperature and fed a chow diet. B: *Scarb1* expression in different tissues of *Scarb1*^*fl/fl*^*-Cdh5*^*Cre+*^ and *Scarb1*^*fl/fl*^*-Cdh5*^*Cre*−^ mice. Gene expression is presented as mean values ± SEM and was normalized for *TATA-box binding protein* (*Tbp*). Different letters indicate statistical difference *P* < 0.05. C: Western blot analysis and (D) quantification of SR-B1 protein expression in different tissues of *Scarb1*^*fl/fl*^*-Cdh5*^*Cre+*^ and *Scarb1*^*fl/fl*^*-Cdh5*^*Cre*−^ mice. Protein expression is presented as mean values ± SEM and was normalized for AKT, and the *Scarb1*^*fl/fl*^*-Cdh5*^*Cre*−^ group was set to 1. Different letters indicate statistical difference *P* < 0.05. E–G: Indirect calorimetry measurements and core body temperature measurements of male *Scarb1*^*fl/fl*^*-Cdh5*^*Cre+*^ (green line) and *Scarb1*^*fl/fl*^*-Cdh5*^*Cre*−^ mice (black line) at an ambient cage temperature of ca. 22°C (red line) were continuously recorded over a period of 3 days. The gray shading marks the lights-off period. (E) Body core temperature, (F) energy expenditure (G), and RER (G) are presented.

To investigate the relevance of endothelial SR-B1 (encoded by *Scarb1*) for lipoprotein processing and adaptive thermogenesis, we crossed mice expressing Cre recombinase under the control of the VE-cadherin (Cdh5) promoter with *Scarb1*^flox/flox^ mice to generate mice lacking SR-B1 in endothelial cells (Cre+) and control littermates (Cre−). To validate the model, we isolated CD31-positive endothelial cells (CD31+), Cd11b-positive myeloid cells (Cd11b+), and mAs from iBAT extracts of chow-fed *Scarb1*^*fl/fl*^*-Cdh5*^*Cre+*^ and *Scarb1*^*fl/fl*^*-Cdh5*^*Cre*−^ mice kept at room temperature by MACS®. The endothelial fraction of *Scarb1*^*fl/fl*^*-Cdh5*^*Cr+*^ mice had substantially lower *Scarb1* expression compared with *Scarb1*^*fl/fl*^*-Cdh5*^*Cre*−^ mice, whereas no significant differences were detected in the other fractions (Fig. 2A). Residual *Scarb1* expression was detected in the endothelial fraction of *Scarb1*^*fl/fl*^*-Cdh5*^*Cre+*^*,* which may be explained by incomplete Cre-mediated gene deletion. Cellular identity of the isolated fractions was confirmed as described above by measuring the cell type-specific markers *Gpihbp1* (Supplemental Fig. S1A), *Emr1* (Supplemental Fig. S1B), and for brown adipocytes *Ucp1* (Supplemental Fig. S1C). *Emr1* and *Ucp1* expression was exclusively expressed in the macrophage and mA fractions, respectively, with no differences between *Scarb1*^*fl/fl*^*-Cdh5*^*Cre+*^ and *Scarb1*^*fl/fl*^*-Cdh5*^*Cre*−^ mice. *Gpihbp1* was detected in both the endothelial fraction as well as the mA fraction, which may be explained by the close interaction between endothelial cells and brown adipocytes, as reviewed (26). This could also account for the slight decrease in *Scarb1* expression in the adipocyte (mA) fraction in *Scarb1*^*fl/fl*^*-Cdh5*^*Cre+*^ mice (Fig. 2A). Notably, an endothelial-specific KO of SR-B1 resulted in reduced *Scarb1* gene and SR-B1 protein expression at the whole-organ level in the heart, iBAT, inguinal WAT (iWAT), gonadal WAT, and skeletal muscle (quadriceps) in *Scarb1*^*fl/fl*^*-Cdh5*^*Cre+*^ mice compared with *Scarb1*^*fl/fl*^*-Cdh5*^*Cre*−^ mice (Fig. 2B–D). No differences in *Scarb1* and SR-B1 expression were observed in the liver or adrenal glands between *Scarb1*^*fl/fl*^*-Cdh5*^*Cr +*^ mice and *Scarb1*^*fl/fl*^*-Cdh5*^*Cre*−^ mice (Fig. 2B–D), as the primary cells expressing SR-B1 in these organs are hepatocytes (27), liver sinusoidal endothelial cells (28) which are not affected by the *Cdh5* KO due to minimal *Cdh5* expression in these cells (29, 30) and the adrenal cells, respectively (31). Overall, these data demonstrated efficient depletion of SR-B1 in heart, adipose tissues, and muscle, indicating that SR-B1 of peripheral metabolically active organs is primarily expressed by vascular endothelial cells.

Next, we characterized baseline physiological parameters and plasma metabolic markers of these mice, which were housed at room temperature and fed a standard chow diet. Significant changes were detected neither in plasma glucose, cholesterol, or triglyceride levels nor in body or organ weights except for an increased liver mass in *Scarb1*^*fl/fl*^*-Cdh5*^*Cre+*^ mice compared with control littermates (Supplemental Fig. S1D–J). To determine energy expenditure and core body temperature, we housed mice in metabolic cages for indirect calorimetry combined with transponder-based temperature monitoring. No differences were observed between *Scarb1*^*fl/fl*^*-Cdh5*^*Cre+*^ and *Scarb1*^*fl/fl*^*-Cdh5*^*Cre*−^ mice at room temperature with respect to body temperature (Fig. 2E), energy expenditure (Fig. 2F), and RER (Fig. 2G), indicating that endothelial SR-B1 expression does not alter systemic energy metabolism under these conditions.

### SR-B1 expressed by endothelial cells is dispensable for adaptive thermogenesis

**Fig. 3:**
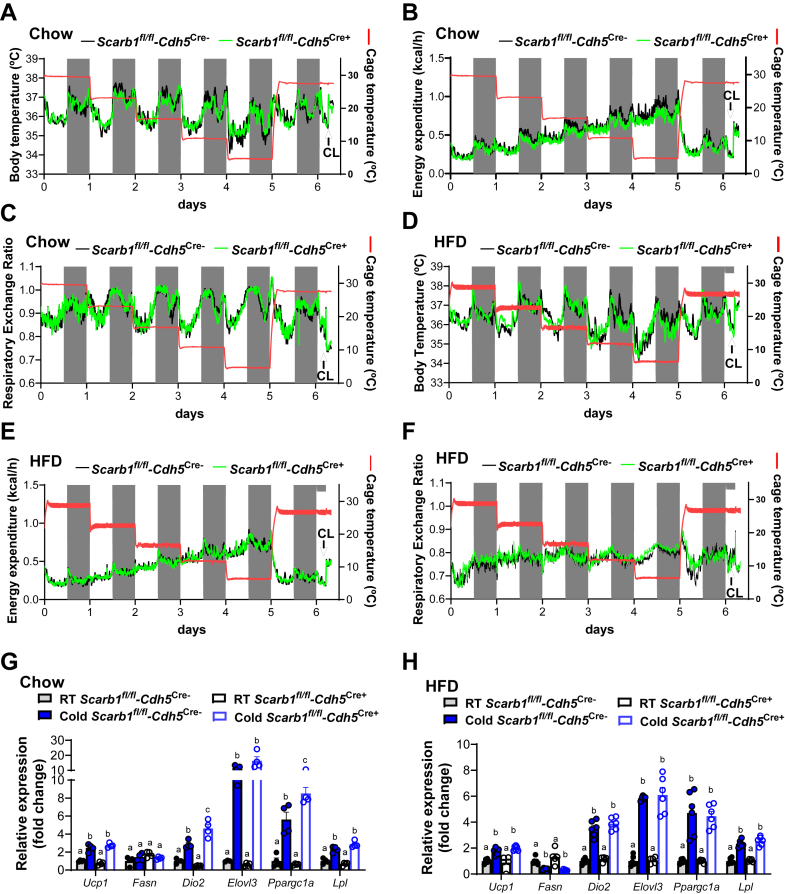
Role of endothelial-specific SR-B1 deletion in response to cold exposure and HFD feeding. A–F: Energy expenditure was determined by indirect calorimetry in male *Scarb1*^*fl/fl*^*-Cdh5*^*Cre+*^ (green line) and *Scarb1*^*fl/fl*^*-Cdh5*^*Cre*−^ mice (black line) fed a (A–C) standard chow or (D–F) an HFD. The gray shading marks the lights-off period, and the ambient housing temperature (red line) was decreased from 30°C to 6°C by step-wise lowering the temperature by 6°C every day over a course of 5 days. Then, ambient temperature was increased to 28°C to inactivate BAT-dependent thermogenesis, and mice received a CL316,243 injection (CL, see arrow) to measure maximal BAT capacity. (A) Body temperature, (B) energy expenditure, and (C) RER of chow-fed mice are presented as a mean (n = 6). D–F: Male *Scarb1*^*fl/fl*^*-Cdh5*^*Cre+*^ and *Scarb1*^*fl/fl*^*-Cdh5*^*Cre*−^ mice were fed an HFD for 4 weeks. (D) Body temperature, (E) energy expenditure, and (F) RER of HFD-fed mice are presented as a mean (n = 6). G: Female *Scarb1*^*fl/fl*^*-Cdh5*^*Cre+*^ and *Scarb1*^*fl/fl*^*-Cdh5*^*Cre*−^ mice on a chow diet were housed at room temperature or 6°C (cold) for 24 h (n = 4). H: Male *Scarb1*^*fl/fl*^*-Cdh5*^*Cre+*^ and *Scarb1*^*fl/fl*^*-Cdh5*^*Cre*−^ mice were fed an HFD for 4 weeks and afterward housed at room temperature or 6°C for 24 h before the oral glucose fat tolerance test (n = 6). G and H: Expression data of metabolic genes were determined in BAT and normalized to *Tbp*. Data are presented as mean values ± SEM, and at room temperature, *Scarb1*^*fl/fl*^*-Cdh5*^*Cre*−^ group was set to 1. Different letters indicate statistical difference *P* < 0.05. *Dio2* (type II iodothyronine deiodinase); *Elovl3* (elongation of very long chain fatty acid 3); *Fasn* (fatty acid synthase); *Ppargc1a* (peroxisome proliferator-activated receptor-gamma coactivator 1a); *Ucp1* (uncoupling protein 1); *Lpl*. Please note that all data points are plotted, but some symbols coincide, which give the impression of a fewer *n* number.

To address the role of endothelial cell-specific SR-B1 expression in whole-body energy balance during adaptation to cold, indirect calorimetry was performed at different housing temperatures in chow-fed *Scarb1*^*fl/fl*^*-Cdh5*^*Cre+*^ and *Scarb1*^*fl/fl*^*-Cdh5*^*Cre*−^ mice. The ambient temperature was gradually reduced from 30°C to 6°C (6°C decrements daily) and then increased to 28°C for 1.5 days. Finally, 3 hours before sacrifice, mice received an injection of the β3-adrenergic receptor agonist CL316,243 to assess maximal BAT capacity. As expected (32), all measured metabolic parameters exhibited a diurnal pattern regardless of temperature (Fig. 3A–C), whereas cold exposure substantially increased energy expenditure (Fig. 3B). However, no significant differences were observed between *Scarb1*^*fl/fl*^*-Cdh5*^*Cre+*^ and *Scarb1*^*fl/fl*^*-Cdh5*^*Cre*−^ mice in body temperature (Fig. 3A), energy expenditure (Fig. 3B), RER (Fig. 3C), body weight, and organ weights (Supplemental Fig. S2A, B).

Next, we challenged lipid metabolic pathways by forcing a switch in energy substrate utilization through feeding a cholesterol-containing HFD. In both genotypes, HFD feeding did not affect body core temperature (Fig. 3D) or total energy expenditure (Fig. 3E), but it abolished the diurnal rhythm of the RER (Fig. 3F), reflecting sustained fat oxidation (RER ∼0.7) (33). However, also under conditions of enhanced lipid handling, no major differences were observed between *Scarb1*^*fl/fl*^*-Cdh5*^*Cre+*^ and *Scarb1*^*fl/fl*^*-Cdh5*^*Cre*−^ mice regarding all these parameters, including body and organ weights (Supplemental Fig. S2C, D). As subtle changes in energy metabolism may not be detectable by indirect calorimetry, we determined the expression of thermogenic and lipid-handling genes in iBAT and iWAT from chow- and HFD-fed *Scarb1*^*fl/fl*^-Cdh5^Cre+^ and *Scarb1*^*fl/fl*^-Cdh5^Cre−^ mice was exposed to 6°C for 24 h or kept at room temperature, as the expression levels of these genes directly reflect the activation status of the tissues. In iBAT, *Ucp1*, *Elovl3*, *Dio2*, *Ppargc1a*, and *Lpl* expression increased upon cold exposure regardless of the genotype and the dietary conditions (Fig. 3G, H). Under conditions of thermogenic activation, chow-fed *Scarb1*^*fl/fl*^*-Cdh5*^*Cr +*^ mice have slightly higher expression of *Dio2* and *Ppargc1a* compared with *Scarb1*^*fl/fl*^*-Cdh5*^*Cre*−^ mice (Fig. 3G), an effect that was blunted under HFD conditions. Cold exposure promotes the appearance of inducible brown (beige) adipocytes in various WAT depots (34), which results in a similar induction of thermogenic gene expression when comparing iWAT and BAT from cold-exposed mice. Accordingly, we observed an increase or trend toward higher expression for *Ucp1*, *Fasn*, *Elovl3*, and *Ppargc1a* upon cold exposure regardless of the genotype and the diet in iWAT (Supplemental Fig. S2E, F). Similar to the data generated in iBAT of chow-fed mice, cold-exposed *Scarb1*^*fl/fl*^*-Cdh5*^*Cre+*^ mice had higher *Dio2* expression compared with *Scarb1*^*fl/fl*^*-Cdh5*^*Cre*−^ mice (Supplemental Fig. S2E). Overall, endothelial SR-B1 appears dispensable for systemic energy expenditure and adaptive thermogenic responses in BAT and WAT under basal and metabolic stress conditions induced by cold exposure or HFD feeding.

### Role of endothelial SR-B1 for lipid and glucose disposal in response to thermogenic activation

**Fig. 4:**
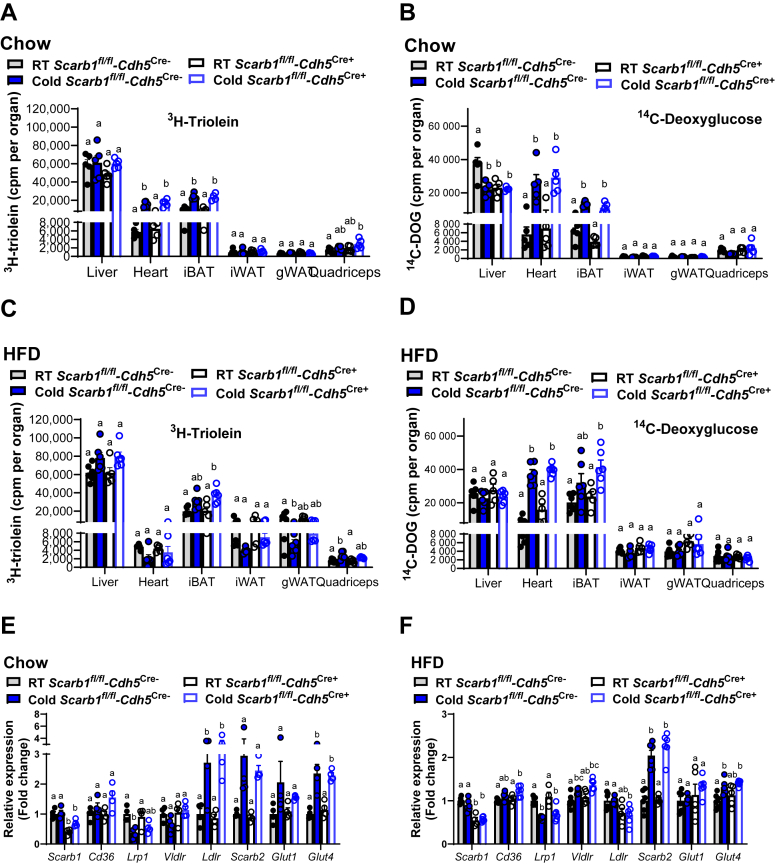
Impact of endothelial SR-B1 on organ-specific glucose and lipid handling in response to cold exposure and HFD feeding. A–D: A postprandial tolerance test was performed by orally applying a glucose-lipid emulsion that was traced with ^3^H-triolein and ^14^C-DOG. Two hours after gavage, mice were sacrificed, and radioactivity was determined in different organs. Data are presented as mean values per organ. Different letters indicate statistical difference *P* < 0.05. A and B: Male *Scarb1*^*fl/fl*^*-Cdh5*^*Cre+*^ and *Scarb1*^*fl/fl*^*-Cdh5*^*Cre*−^ mice on a chow diet were housed at room temperature or 6°C for 24 h before the postprandial challenge (n = 5). Uptake of (A) TRL-derived ^3^H-labeled triolein/oleic acid and (B) ^14^C-DOG by different organs. C, D, F: Male *Scarb1*^*fl/fl*^*-Cdh5*^*Cre+*^ and *Scarb1*^*fl/fl*^*-Cdh5*^*Cre*−^ mice were fed an HFD for 4 weeks and afterward housed at room temperature or 6°C for 24 h before the oral glucose fat tolerance test (n = 6). Uptake of (C) TRL-derived ^3^H-labeled triolein/oleic acid and (D) ^14^C-DOG. E: Female *Scarb1*^*fl/fl*^*-Cdh5*^*Cre+*^ and *Scarb1*^*fl/fl*^*-Cdh5*^*Cre*−^ mice were housed at room temperature or 6°C (cold) for 24 h (n = 4) and fed a chow diet. E and F: Expression data of lipoprotein receptors and glucose transporters were determined in BAT isolated from chow (E) or (F) HFD-fed mice and normalized to *Tbp*. Data are presented as mean values ± SEM, and room temperature *Scarb1*^*fl/fl*^*-Cdh5*^*Cre*−^ group was set to 1. Different letters indicate statistical difference *P* < 0.05. *Cd36* (cluster of differentiation 36); *Glut1* (glucose transporter 1), *Glut4* (glucose transporter 4); *Ldlr* (low-density lipoprotein receptor); *Lrp1* (LDL receptor-related protein-1); *Scarb1* (scavenger receptor class B1); *Scarb2* (scavenger receptor class B2, also known as lysosomal integral membrane protein 2); *Vldlr* (very-low-density lipoprotein receptor). Please note that all data points are plotted, but some symbols coincide, which gives the impression of a fewer n number.

To investigate the potential relevance of endothelial SR-B1 for organ-specific handling of energy substrates, we performed combined oral glucose fat tolerance tests in chow-fed *Scarb1*^*fl/fl*^*-Cdh5*^*Cre+*^ and *Scarb1*^*fl/fl*^*-Cdh5*^*Cre*−^ mice housed at 6°C for 24 h or at room temperature. The lipid emulsions were traced with ^3^H-triolein and ^14^C-deoxyglucose (^14^C-DOG) to quantify the organ-specific uptake 2 hours after gavage. In the heart and iBAT of chow-fed mice, and regardless of genotype, cold exposure substantially increased the uptake of both TRL-derived ^3^H-labeled triolein/oleic acid and ^14^C-DOG uptake (Fig. 4A, B). Interestingly, livers of *Scarb1*^*fl/fl*^*-Cdh5*^*Cre*−^ mice kept at room temperature had higher uptake of ^14^C-DOG compared with cold-exposed mice and *Scarb1*^*fl/fl*^*-Cdh5*^*Cre+*^ mice kept at room temperature (Fig. 4B), whereas no other differences were observed (Fig. 4A, B). Aside from the cold-induced lowering of plasma triglycerides compared with *Scarb1*^*fl/fl*^*-Cdh5*^*Cre*−^ mice kept at room temperature (Supplemental Fig. S3D), no major differences in body characteristics were observed between chow-fed *Scarb1*^*fl/fl*^*-Cdh5*^*Cre+*^ mice and *Scarb1*^*fl/fl*^*-Cdh5*^*Cre*−^ mice (Supplemental Figs. S3A–C and S4E). In a similar setup, we repeated the oral glucose fat tolerance test experiment in mice that were fed an HFD for 4 weeks. In iBAT and skeletal muscle, there was a trend toward increased uptake of TRL-derived ^3^H-labeled triolein/oleic acid upon cold exposure regardless of the genotype (Fig. 4C). Notably, in contrast to the results obtained under chow diet conditions, we observed lower TRL-derived ^3^H-labeled triolein/oleic acid (Fig. 4C) and a shift toward higher ^14^C-DOG uptake (Fig. 4D). However, no major differences in energy substrate uptake, body characteristics, and plasma parameters when comparing HFD-fed *Scarb1*^*fl/fl*^*-Cdh5*^*Cre+*^ mice with *Scarb1*^*fl/fl*^*-Cdh5*^*Cre*−^ were observed (Fig. 4C, D, Supplemental Fig. S3F–J). To determine whether the lack of differences can be explained by a compensatory upregulation of glucose transporters and other lipoprotein receptors, we examined receptor gene expression in iBAT (Fig. 4E, F) and iWAT (Supplemental Fig. S3K, L). In both iBAT and iWAT, *Scarb1* expression was decreased in *Scarb1*^*fl/fl*^*-Cdh5*^*Cre+*^ mice irrespective of environmental conditions and diet compared with *Scarb1*^*fl/fl*^*-Cdh5*^*Cre*−^ mice (Fig. 4E, F, Supplemental Fig. S3K, L). Aside from the cold-triggered increase in *Ldlr*, *Glut4*, *and Scarb2* expression in iBAT of chow-fed and/or HFD-fed mice, no main differences in the expression of other genes investigated were observed. Thus, we were not able to obtain evidence for a lipoprotein receptor that could compensate for the loss of endothelial SR-B1, at least on the basis of the targeted gene expression analysis. Overall, these data indicate that endothelial SR-B1 is dispensable for glucose and lipid uptake, regardless of diet or thermogenic activation.

### Endothelial SR-B1 promotes selective cholesterol uptake into the heart and BAT

**Fig. 5:**
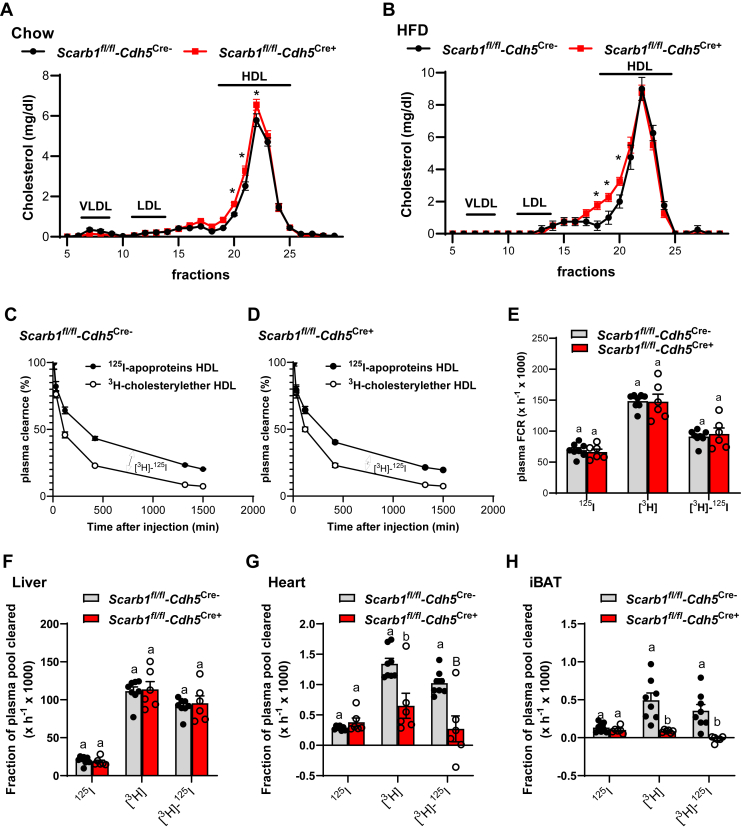
Endothelial SR-B1 promotes selective cholesterol uptake in heart and BAT. A and B: For FPLC analysis, individual plasma samples were analyzed, and cholesterol levels were determined in each fraction (n = 4 per group). Cholesterol profiles of plasma obtained from male *Scarb1*^*fl/fl*^*-Cdh5*^*Cre+*^ and *Scarb1*^*fl/fl*^*-Cdh5*^*Cre*−^ mice on (A) a chow diet or on a (B) HFD diet are presented. C–F: Male *Scarb1*^*fl/fl*^*-Cdh5*^*Cre+*^ and *Scarb1*^*fl/fl*^*-Cdh5*^*Cre*−^ mice on a chow diet were housed at room temperature (n = 8). For metabolic turnover, mice were intravenously injected with HDL double-labeled with ^125^I-apolipoproteins and ^3^H-cholesteryl ether. Plasma samples were isolated at indicated time points. After 24 h, organs were harvested, and radioactivity was determined in plasma samples, liver, heart, and BAT. C–E: FCR rates were calculated and presented as the mean for ^125^I-apolipoproteins, and ^3^H-cholesteryl ether in the plasma of (C) *Scarb1*^*fl/fl*^*-Cdh5*^*Cre*−^ and (D) *Scarb1*^*fl/fl*^*-Cdh5*^*Cre+*^ mice over a course of 24 h. E: FCR plasma rates are presented as the mean values for ^125^I-apolipoproteins, ^3^H-cholesterol ether, and the difference indicates selective clearance of cholesterol ether. The fractions of ^125^I-apolipoproteins, ^3^H-cholesterol ether, and selective ^3^H-cholesterol ether cleared by (F) the liver, (G) the heart, and (H) the BAT are presented as mean values ± SEM. Different letters indicate statistical difference *P* < 0.05.

Given the established role of SR-B1 in reverse cholesterol transport, selective cholesteryl ester uptake, and HDL metabolism in hepatocytes (18), we investigated whether SR-B1 specifically contributes to cholesterol uptake via HDL in endothelial cells. As global SR-B1 deficiency is associated with a substantial increase in HDL cholesterol (23, 35), we first performed lipoprotein profiling of individual plasma samples obtained from *Scarb1*^fl/fl^-Cdh5Cre^−^ and *Scarb1*^fl/fl^-Cdh5Cre^+^ mice that were fed either a standard chow (Fig. 5A) or an HFD (Fig. 5B) by FPLC. Under both dietary conditions, mice lacking endothelial SR-B1 expression exhibited slightly elevated cholesterol levels in the HDL fractions. Next, we investigated HDL clearance by injecting radioactively double-labeled HDL particles (^125^I HDL apolipoprotein and ^3^H-cholesteryl ether) into chow-fed *Scarb1*^*fl/fl*^*-Cdh5*^*Cre*−^ and *Scarb1*^*fl/fl*^*-Cdh5*^*Cre+*^ mice housed at room temperature. No differences in plasma-FCR were observed between *Scarb1*^*fl/fl*^*-Cdh5*^*Cre*−^ and *Scarb1*^*fl/fl*^*-Cdh5*^*Cre+*^ mice (Fig. 5C–H). Similarly, we did not detect differences between the genotypes with regard to selective cholesteryl ether uptake in the liver, which is the main organ for clearance of HDL-derived cholesterol (Fig. 5F) (36, 37), or other organs, such as kidney, intestine, or spleen (Supplemental Fig. S4A–C). Notably, in both the heart and BAT, we observed an almost complete reduction in selective uptake of cholesteryl ether from HDL particles in *Scarb1*^*fl/fl*^*-Cdh5*^*Cre+*^ mice compared with *Scarb1*^*fl/fl*^*-Cdh5*^*Cre*−^ mice (Fig. 5G, H). To our knowledge, these findings show for the first time that endothelial SR-B1 facilitates selective cholesterol uptake in BAT and the heart.

## Discussion

SR-B1 is a key player in reverse cholesterol transport and HDL metabolism (18, 38, 39). The importance of SR-B1 in reverse cholesterol transport has been demonstrated in whole-body SR-B1 KO (40) and liver-specific KO (41) mice, which exhibit large, cholesterol-enriched HDL particles, elevated plasma cholesterol levels, and an increased risk of atherosclerosis (42, 43). These effects are also observed in humans carrying an SNP in the *SCARB1* gene, which is associated with increased HDL cholesterol levels and an elevated risk of coronary heart disease (44, 45, 46, 47). The function of SR-B1 varies depending on the cell type in which it is expressed. In hepatocytes and adrenal cells, SR-B1 extracts cholesteryl esters from HDL particles without internalizing the particle (39, 48), whereas in the peripheral tissues, SR-B1 is involved in cholesterol efflux from macrophages (49). A large cavity that spans the entire SR-B1 protein appears to facilitate this bidirectional flux of cholesterol (50). As reviewed (38), the central dogma of reverse cholesterol transport posits that ABCA1 and ABCG1 transporters mediate cholesterol efflux from peripheral tissues and macrophages to pre-β or nascent HDL, which then travels to the liver. There, SR-B1 extracts the cholesteryl esters from mature HDL particles. The resulting lipid-poor HDL particles can re-enter circulation, whereas the extracted cholesteryl esters may be used for bile synthesis or be excreted. Reverse cholesterol transport is especially important for peripheral cells, as they lack intrinsic mechanisms to eliminate excess cholesterol, and accumulation of cholesterol can be cell toxic.

In order for HDL to reach the parenchymal cells, it must cross the endothelial barrier. HDL transcytosis in microvascular endothelial cells is SR-B1 dependent (51), whereas in aortic endothelial cells, HDL transcytosis depends on SR-B1 and ABCG1 (52). Besides mediating cholesterol levels, HDL also has other beneficial effects. For example, it was shown in vitro that the beneficial properties of HDL—such as endothelial cell migration (53), re-endothelization (54), anti-inflammatory effects, and maintenance of endothelial integrity via activation of endothelial nitric oxide synthase (53, 55, 56)—are mediated by SR-B1. Beyond its role in HDL metabolism, endothelial SR-B1 has been implicated in LDL transcytosis (19, 57), and previous studies have demonstrated that SR-B1 can bind various TRL particles as well (58, 59, 60). Given that SR-B1 *i*) can bind different TRL particles, *ii*) is highly expressed in the endothelial fraction of BAT and WAT in mice and humans, *iii*) belongs to the same receptor family as CD36 (11), *iv*) is known to be important for thermogenesis (7), and *v*) endothelial cells are critical for BAT function (16), we hypothesized that endothelial SR-B1 contributes to adaptive thermogenesis and lipid uptake into thermogenic tissues. We found that endothelial SR-B1 contributes to the majority of whole organ SR-B1 expression in heart, skeletal muscle, and adipose tissue depots, with highest expression in BAT. However, while we cannot rule out the possibility that residual SR-B1 activity in the tissues is sufficient to fulfil its functional roles, we demonstrate that endothelial SR-B1 is not essential for thermogenesis, fatty acid, or glucose uptake, regardless of diet or ambient temperature. Moreover, the nadir respiratory-exchange ratio (≈0.70) during cold or β_3_-adrenergic stimulation was similar between the genotypes studied, suggesting that both fatty-acid oxidation in BAT and fatty acid release regulated by intracellular lipases in WAT are most likely similar between control and endothelial-specific SR-B1 KO mice. Aside from the increased expression of some thermogenic genes in iBAT of chow-fed cold-exposed mice lacking endothelial SR-B1, we did not observe an altered expression of thermogenic markers or lipoprotein receptors that could compensate for the loss of endothelial SR-B1. The most likely explanation for the lack of metabolic phenotype in endothelial SR-B1-deficient mice is that CD36 remains present and, as a long-chain fatty acid transporter, continues to mediate substantial lipid uptake (5). To test this hypothesis directly, the time-consuming and costly generation of endothelial-specific SR-B1 KO mice on a CD36-deficient background would be required. Another compensatory receptor could be the VLDLR that has been shown to transport VLDL-derived fatty acids to brown adipocytes for beta oxidation and activation of the transcription factor peroxisome proliferator-activated receptor delta (25). However, in the current study, we show that the VLDLR is predominantly expressed by parenchymal adipocytes rather than by vascular endothelial cells, suggesting that the potential effect on thermogenesis and triglyceride metabolism is masked by CD36 or other lipid/lipoprotein receptors expressed by endothelial cells, or even by residual SR-B1 expression detected in the mouse model. It is interesting to note that despite the observed higher expression of the LDL receptor in response to cold exposure, this lipoprotein receptor is dispensable for the accelerated lipoprotein processing in thermogenic adipose tissues in response to cold exposure (7). Another member of the LDL receptor family, the multifunctional LDL receptor-related protein-1 (LRP1), has been shown to be a critical regulator of adipose tissue biology. Notably, the adipocyte-specific deletion of LRP1 resulted in an impaired clearance of postprandial TRLs and lower adipose tissue mass, which is associated with a compensatory elevation of energy expenditure in muscle thermogenesis (61). On the other hand, endothelial-specific LRP1 KO mice display improved lipid profiles and have higher energy expenditure (62), emphasizing the relevance of lipoprotein receptors expressed by endothelial cells in lipid handling and systemic energy homeostasis. Interestingly, both adipocyte- and endothelial-specific LRP1 KO mice are characterized by an improved glucose tolerance (61, 62), indicating a regulatory axis between lipid and glucose uptake by adipose tissue. For SR-B1, it has been reported that the siRNA-mediated knockdown resulted in lower glucose uptake in 3T3-L1 adipocytes (63). In line, adipocyte-specific deletion of SR-B1 blunted insulin-stimulated glucose uptake (64). In our studies, glucose tracer uptake into adipose tissues and whole-body glucose tolerance were unaffected, suggesting the endothelial SR-B1 is at least not essential for the tissue-specific glucose handling.

The most important result of the present study was that we showed that mice without endothelial SR-B1 had lower selective cholesterol uptake in BAT and the heart. As far as we know, this has not been reported before. The influx of cholesterol via SR-B1 has been primarily studied in the liver and steroidogenic organs (49, 65, 66), and HDL processing by endothelial cells has largely been investigated in cell culture systems (51, 52, 57). However, almost 40 years ago, it was already shown that endothelial cells are capable of taking up cholesterol from HDL particles (66). More recently, a study demonstrated that arterial endothelial cells in the placenta, which express higher levels of SR-B1 than venous endothelial cells, exhibited increased selective cholesteryl ester uptake (67). Consistent with these in vitro findings, our data clearly demonstrate that SR-B1 expressed by endothelial cells contributes significantly to whole organ expression in BAT and the heart and that it extracts cholesteryl esters from HDL in a directional manner. Although ^125^I-TC is considered a robust marker for holoparticle uptake, a minor fraction of the protein label could dissociate from HDL (68), which would, however, rather lead to an underestimation of tissue apolipoprotein uptake.

The physiological relevance of SR-B1-dependent cholesterol delivery to metabolically active BAT and heart remains elusive. Recently, it has been shown that the ratio of glycerophospholipids to cholesterol in mitochondrial membranes differs substantially between different organs and that an increase in mitochondrial cholesterol content impairs UCP1 activity in brown adipocytes (69). In the heart, transendothelial transfer of cholesterol could *i*) furnish cardiomyocytes with unesterified sterol for continuous sarcolemmal and T-tubule membrane renewal, *ii*) spare these cells the energetic cost of de novo synthesis, and *iii*) operate in concert with SR-B1-dependent nitric oxide signaling to preserve coronary microvascular reactivity. In line, recent studies demonstrated that cardiomyocyte cholesterol depletion compromises β-oxidation and contractile performance, further underscoring the potential importance of SR-B1-dependent cholesterol delivery via the endothelium in sustaining myocardial energetics and mitochondrial function (70, 71, 72). However, at least on the systemic level, we did not detect differences in energy expenditure when comparing control versus endothelial-specific SR-B1 KO on a standard diet or a cholesterol-enriched HFD when exposed to warm or cold ambient temperatures. In this context, it is of note that we detected a higher cold-induced expression of the third member of the CD36 family, the lysosomal integral membrane protein-2 (also known as SCARB2), which has been reported to mediate cholesterol export from lysosomes (73). Altogether, these findings could imply a potential cholesterol transport route that involves all three CD36 family members, where *i*) CD36 facilitates luminal lipoprotein processing, *ii*) SR-B1 mediates transendothelial cholesterol transport, and *iii*) lysosomal integral membrane protein-2 is involved in the subsequent endolysosomal cholesterol handling in adipocytes. Thus, future studies will be important to investigate how endothelial SR-B1-mediated selective cholesterol uptake contributes to organ-specific cholesterol homeostasis in the BAT and the heart.

In conclusion, we showed that despite its high expression level, SR-B1 in endothelial cells of BAT and the heart is not required for adaptive thermogenesis or clearance of energy substrates. However, endothelial SR-B1 mediates selective cholesterol uptake in iBAT and the heart. The functional relevance of this process, for example, in regulating organ-specific cholesterol homeostasis or influencing endothelial cell biology, remains to be elucidated.

## Data availability

All study data are included in the article and supplemental data.

## Supplemental data

This article contains supplemental data.

## Conflict of interest

The authors declare that they have no conflicts of interest with the contents of this article.
