# Supplementary figures and images for: Endothelial SR-B1 is dispensable for thermogenesis but promotes selective cholesterol uptake in brown adipose tissue
Source: J Lipid Res. 2025 Sep 3;66(10):100894. doi: 10.1016/j.jlr.2025.100894 (PMC12510209; doi:10.1016/j.jlr.2025.100894)

Suppl. Figure 1

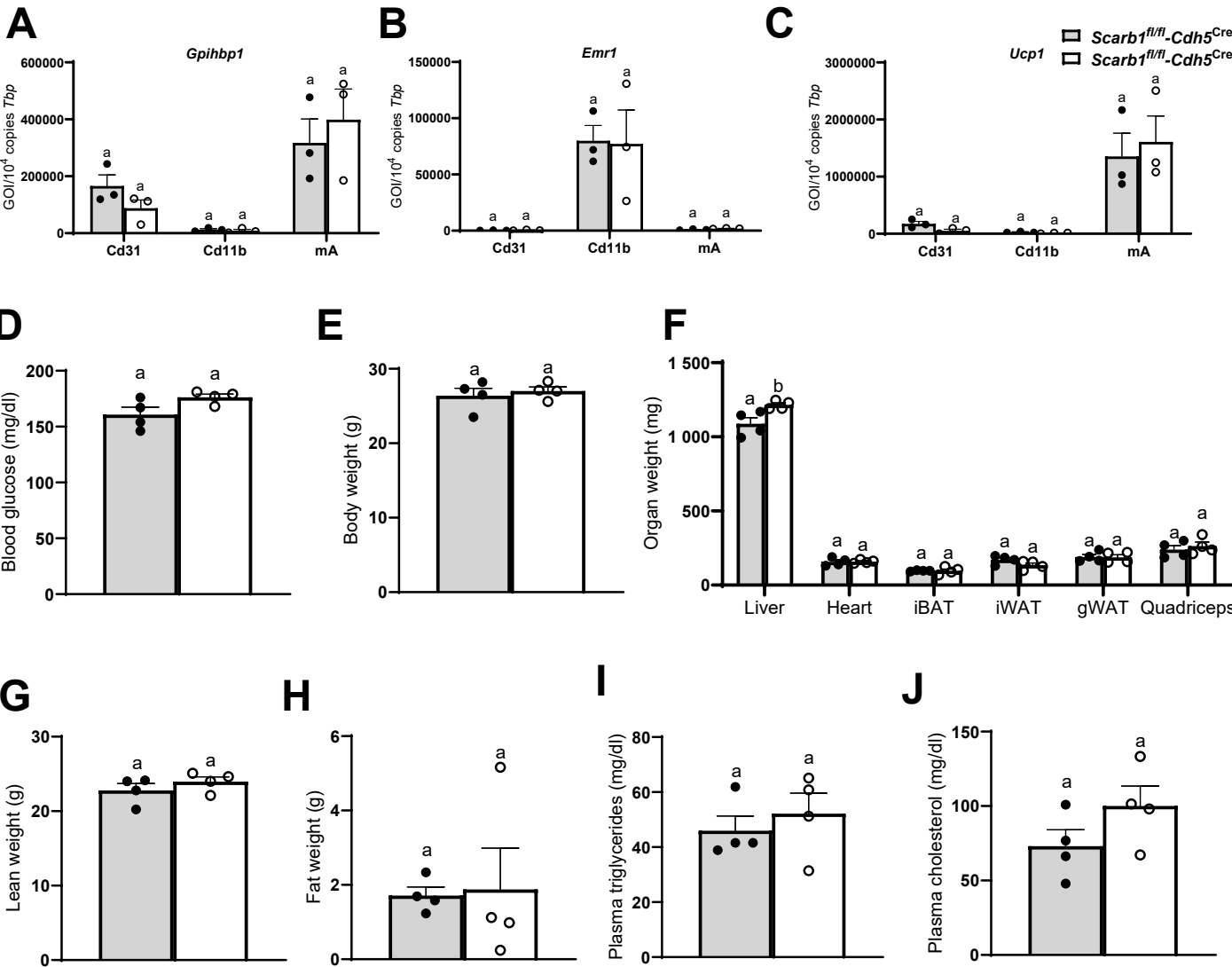

Suppl. Figure 2

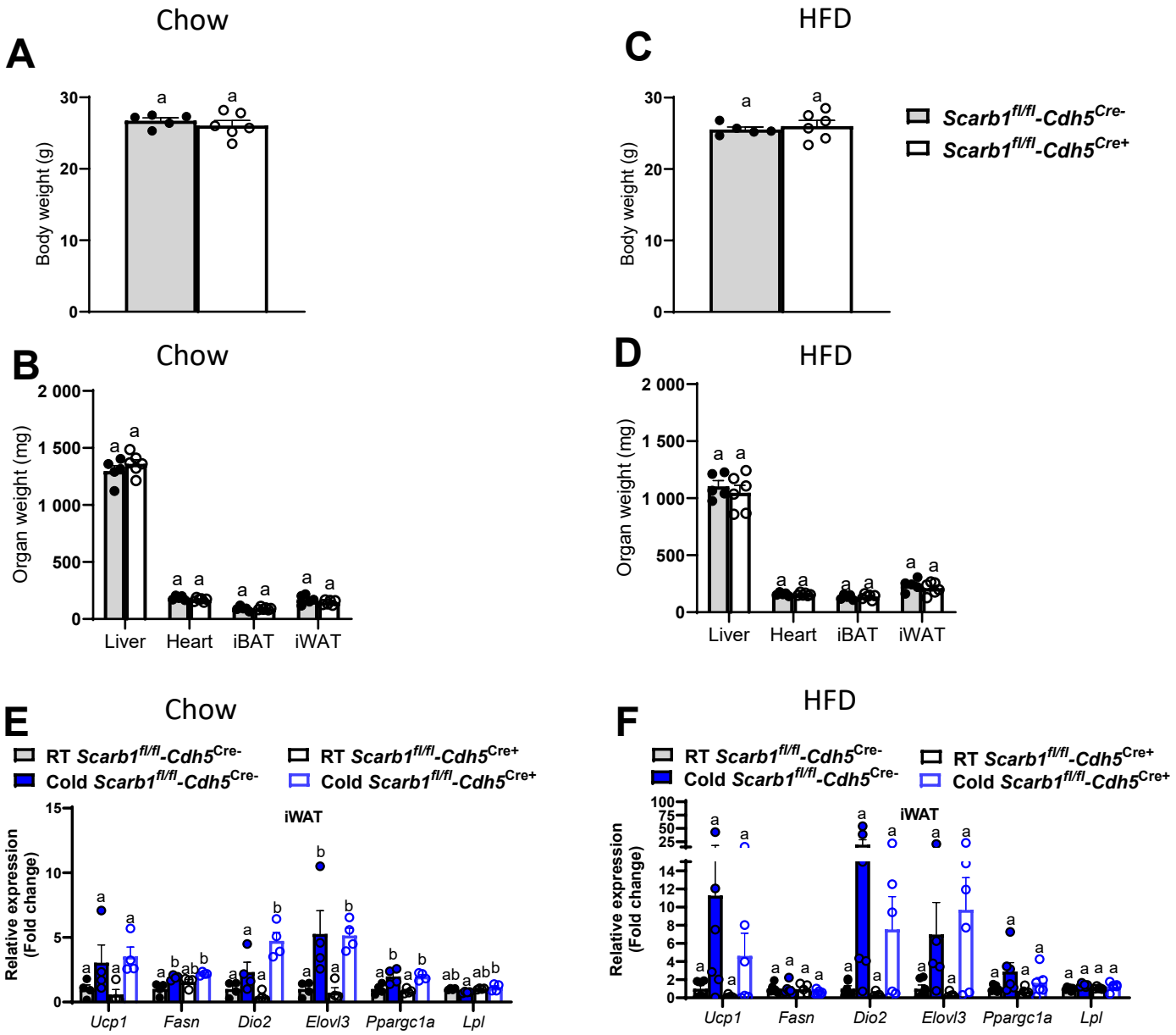

Suppl. Fig. 3

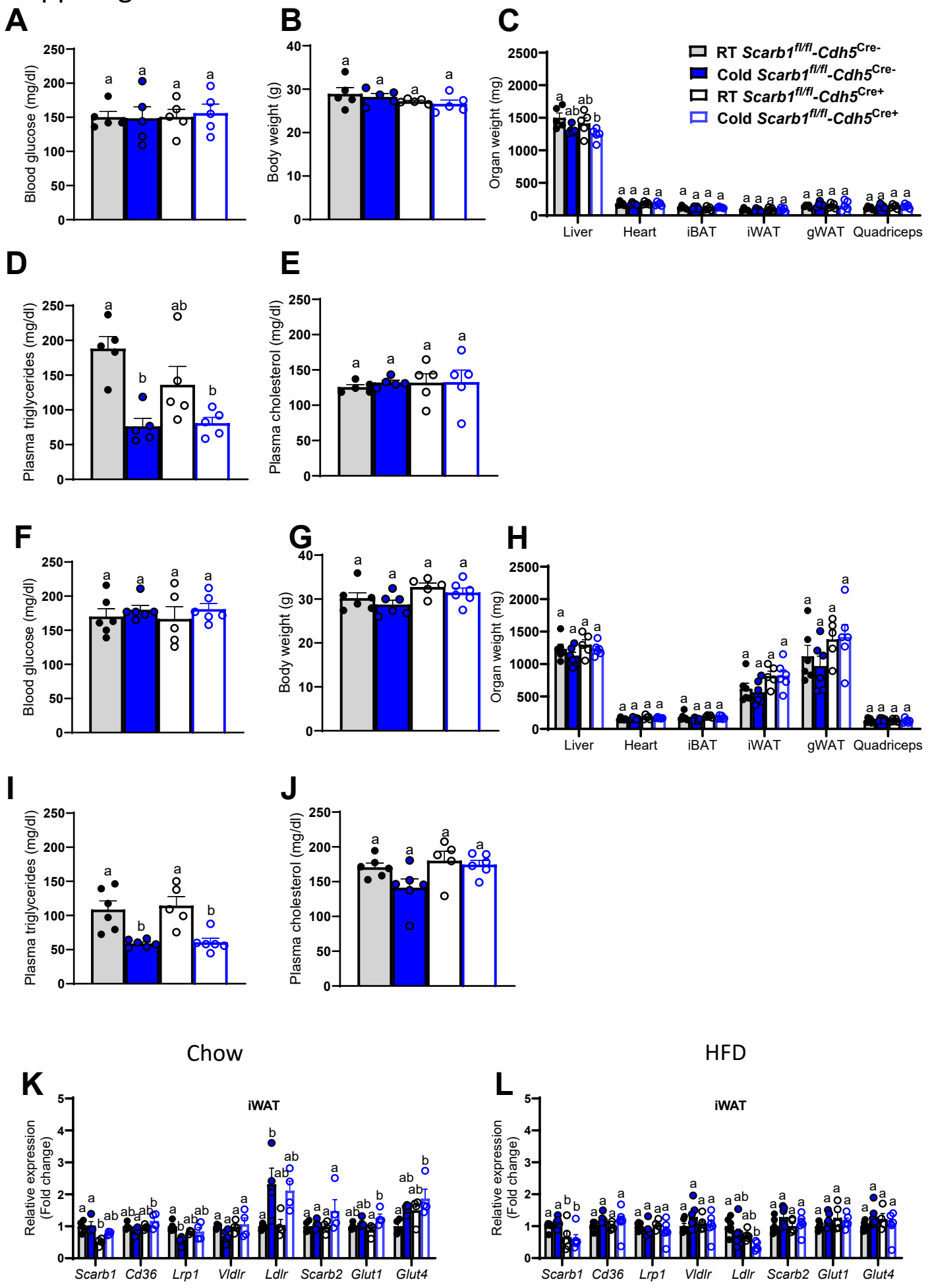

Suppl. Fig. 4

**A**

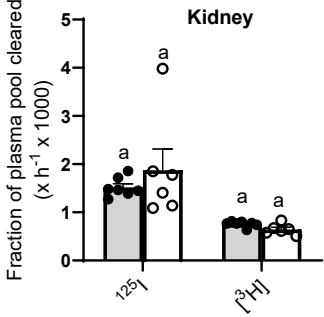

**B**

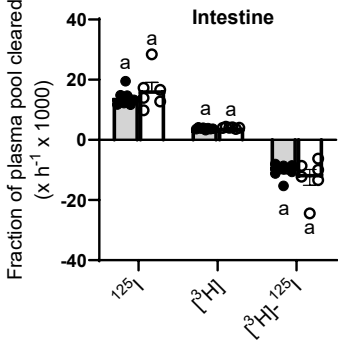

**C**

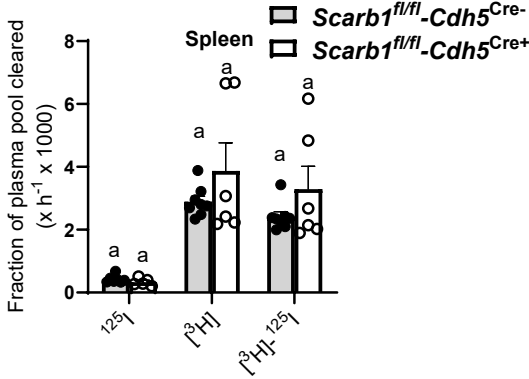

Supplement: Supplementary Figures [file mmc1.pdf]
